# A Current Perspective on Leishmaniasis

**DOI:** 10.4103/0974-777X.62863

**Published:** 2010

**Authors:** Angela Clem

**Affiliations:** *Department of Global Health, MDC 56 College of Public Health, 12901 Bruce B Downs Blvd, Tampa, USA*

**Keywords:** Cutaneous leishmaniasis, Visceral leishmaniasis, Treatments, Vaccines

## Abstract

There are many challenges facing the successful control and eradication of cutaneous and visceral leishmaniasis. Leishmaniasis is still endemic in many poverty stricken and war torn areas. Through the use of an extensive literature review, this article examined the global disease burden of cutaneous and visceral leishmaniasis. Surveillance and control measures for leishmaniasis being used by the World Health Organization were also discussed in this article. Finally, potential new treatments and possible vaccines for leishmaniasis were reviewed in this article.

The articles in the symposium provide a current perspective on the global disease burden of leishmaniasis. These articles also cover control and surveillance measures being used for leishmaniasis and new research on leishmaniasis. This cutting edge research includes potential new treatments as well as possible vaccines for leishmaniasis.

Leishmaniasis is a parasitic disease caused by obligate intracellular protozoans from the genus Leishmania. *Leishmania donovani* and *L. infantum* /*L. chagasi* are commonly the species that are implicated in the disease. These protozoans are typically spread through the bite of infected female phlebotomine sand flies; although congenital and blood-borne transmission has been documented. Leishmaniasis typically presents as one of the two forms, either cutaneous or visceral. Cutaneous leishmaniasis produces skin ulcerations with raised borders which may last months to years and typically result in severe scarring.[1] In contrast, visceral leishmaniasis affects the internal organs especially the spleen, liver, and bone marrow. Latent cases may remain undiagnosed from years to decades until the individual becomes immunocompromised (especially with HIV) then the individual will typically develop fever, weight loss, hepatosplenomegaly, and pancytopenia. Severe cases of visceral leishmaniasis left untreated are commonly fatal.[2] Although there are treatments for leishmaniasis (such as liposomal amphotericin B for visceral leishmaniasis and pentavalent antimonial compound sodium stibogluconate (Pentostam) for cutaneous leishmaniasis), there are no preventive medications or vaccinations for the disease.[1,2]

Leishmaniasis has been greatly underreported for innumerable years. This underestimation of the true health burden of leishmaniasis is due to several reasons. First, reporting of the disease is only mandatory in 32 of the 88 countries affected by leishmaniasis. The WHO has estimated that 2 million new cases occur yearly (1.5 million for cutaneous leishmaniasis and 500,000 for visceral leishmaniasis) and that 12 million people are infected globally.[3]

Next, because leishmaniasis is a disease of poverty, which produces high morbidity but low mortality and results in stigma-producing deformities, it is a disease that is typically kept hidden by the affected individuals and their families. In addition, economic troubles, civil war, and drought with its accompanying starvation have contributed greatly to the spread of leishmaniasis and the continued underreporting of the actual impact of the disease. For example, an epidemic of visceral leishmaniasis was carried into Eritea and Ethiopia in 1997 from neighboring Sudan by migrating farm laborers and refugees fleeing civil unrest in Sudan. Sudan had previously been experiencing a decade-long epidemic of visceral leishmaniasis due to continuing malnutrition and civil unrest.[3]

## LEISHMANIASIS SURVEILLANCE AND CONTROL

The World Health Organization (WHO) network for leishmaniasis surveillance and control aims to reduce the incidence of disease so that these surveillance and control measures can be integrated into each country's health development activities. This network has been extended to include six new institutions from Sudan, Brazil, China, India, Nepal, and Kenya. The overall goals of this network include early diagnosis and treatment including coinfections with HIV, control of sand fly populations through residual insecticide spraying in houses and insecticide-impregnated bed nets, health education and training, and control of epidemics.[4]

As for ongoing research, the WHO has noted improvements with the direct agglutination test with freeze-dried antigen and antigen detection from urine using dipsticks K39/K26. The dipstick K39 was used successfully for serological diagnosis in Sudan, Nepal, India, and Ethiopia. Also, there are ongoing programs for comparing diagnostic testing for visceral leishmaniasis in the field. Also, insecticide-impregnated bed nets were used successfully in Syria to produce a 50% reduction in cutaneous leishmaniasis. In March 2002, India registered the first oral drug, miltefosine, for the treatment of visceral leishmaniasis. The drug has shown cure rates of up to 98%, does not require refrigeration, and can be used to treat cases resistant to conventional antimony therapy; however, there are some potential gastrointestinal side effects, and the medication is a potential teratogen.[5]

More recent research has examined potential new medications for the treatment of leishmaniasis or potential vaccines for the disease. Wang *et al.* studied the use of arylimidamides, DB745 and DB 766, as potential oral treatments for *L. donovani* axenic amastigotes, and intracellular Leishmania. Both compounds produced dose-dependent inhibition of liver parasitemia in mice and hamster models. DB766 was also shown to reduce parasitemia in the spleen and bone marrow in the hamster model.[6]

Research by Banerjee *et al*. studied the relationship of membrane fluidity of antigen-presenting cells (APCs) with defective cell-mediated immunity in visceral leishmaniasis. Using a Leishmania donovani-infected hamster model, the authors found that systemic administration of cholesterol liposomes cured the infection. The cholesterol liposomes corrected the decreased membrane cholesterol of the APCs which allowed the APCs to properly stimulate T-cells and clear the infection.[7]

Mendez *et al*. examined the effect of pyrazinamide on the survival of leishmania major promastigotes and amastigotes. The promastigotes were more sensitive than the amastigotes to the drug. With prolonged treatment, there was increased parasite elimination. It was also noted that the pyrazimamide produced collateral immunostimulation and may be a potential antileishmanial therapy.[8]

Serrano-Martín *et al*. studied the effects of combining amiodarone and miltefosine against *Leishmania mexicana* promastigotes and intracellular amastigotes. The authors had previously demonstrated that the antiarrhythmic medication amiodarone disrupted the intracellular Ca^2+^ homeostasis of the parasites thus inhibiting their *de novo* sterol biosynthesis. In this study, the authors found that miltefosine also disrupted the intracellular Ca^2+^ homeostasis of the parasites. Synergistic use of amiodarone and miltefosine affected the proliferation of intracellular amastigotes inside macrophages and led to a 90% cure in a mouse model.[9]

Recent research has also examined the cellular and humoral immune responses of healed cutaneous leishmaniasis and visceral leishmaniasis. The study examined the effects of two virulence proteins, L. major protein disulfide isomerase (LmPDI) and mitogen-activated protein kinase kinase (MAPKK) on immune responses. MAPKK was found to induce elevated peripheral blood mononuclear cell proliferation, elevated gamma interferon production, and antibody responses. In the study authors theorized that these results may be useful for leishmaniasis vaccination and serodiagnosis.[10]

In summary, there are many challenges facing the successful control and eradication of cutaneous and visceral leishmaniasis. Leishmaniasis is still endemic in many poverty stricken and war torn areas. However, there is continuing research into the treatment of leishmaniasis and potentially vaccinations for the disease.
